# Interactive Effects of Early Exclusive Breastfeeding and Pre-Pregnancy Maternal Weight Status on Young Children’s BMI – A Chinese Birth Cohort

**DOI:** 10.1371/journal.pone.0144357

**Published:** 2015-12-07

**Authors:** Hong Mei, Bingbing Guo, Baomin Yin, Xiong Liang, Linda Adair, Amanda Thompson, Jianduan Zhang

**Affiliations:** 1 Department of Maternal and Child Health, School of Public Health, Tongji Medical College, Huazhong University of Science and Technology, Wuhan, Hubei, China; 2 The Maternal and Child Health Hospital of Zhuhai City, Zhuhai, Guangdong, China; 3 The People’s Hospital of Zhuhai City, Zhuhai, Guangdong, China; 4 Department of Nutrition, University of North Carolina, Chapel Hill, North Carolina, United States of America; 5 Department of Anthropology, University of North Carolina, Chapel Hill, North Carolina, United States of America; Indiana University, UNITED STATES

## Abstract

**Objectives:**

To assess if the maternal pre-pregnancy weight status (MPWS) alters the association of early infant feeding pattern (at one and third months) with infant body mass index (BMI) in the first two years of life.

**Methods:**

A cohort of 2,220 neonates were recruited in a community-based study conducted in China. Body weight and length were measured at birth, at age one and two, with BMI calculated accordingly. The BMI z-scores (BMI-Z) were computed according to the World Health Organization Growth Standard (2006). Feeding patterns were classified as exclusive breastfeeding (EBF), mixed feeding (MF), and formula feeding (FF). General linear models (GLM) were employed to estimate main and interaction effects of EBF and MPWS on children’s BMI-Z.

**Results:**

No main effect of MPWS was found on child BMI-Z at ages one and two, nor the feeding patterns. An interaction between MPWS and feeding patterns was detected (*p*<0.05). For children who were formula fed during the first month, those who were born to overweight/obesity (OW/OB) mothers had a significantly greater BMI-Z at ages one and two, compared with those with underweight/normal weight (UW/NW) mothers. FF children had greater BMI-Z at ages one and two compared with their EBF and MF counterparts, when they were born to OW/OB mothers.

**Conclusions:**

Maternal pre-pregnancy weight control and early initiation of EBF for children are essential for healthy development in children’s BMI, hence the prevention of early life obesity.

## Introduction

Growth is an important indicator for health in infants, and their body size and growth may have lasting effects on health of their adulthood [1]. Proper feeding practices during infancy are essential for attaining and maintaining proper nutrition, health, and development of infants and children [2], and should therefore be a high priority when considering infant growth [3]. The World Health Organization (WHO) and the American Academy of Pediatrics recommend exclusive breastfeeding (EBF) for the first six months of life to achieve optimal growth, development, and health [4, 5]. In high-income regions breastfed infants compared to formula-fed, were found to grow slower, but at a more desirable growth rate up to 2 years of age [6–9], indicating a lower risk of overweight/obesity (OW/OB) of the breastfed infants [4, 10]. This benefit of breastfeeding might be due to a lower level of calorie intake in breastfed infants than formula-fed ones, as the breastfed infants were found to have a better self-regulation in milk intake, and human milk has a more suitable amount of calorie and protein concentration than formula [11]. However, reports about the influence of EBF on growth are not consistent in studies [5–9, 12].

Body size at birth and infant postpartum growth are also influenced by maternal pre-pregnancy weight status (MPWS) [13, 14]. A recent systematic review and meta-analysis indicated that pre-pregnancy OW/OB increases the risk of high birth weight, macrosomia and in subsequent offspring OW/OB [14]. The MPWS influences the development of the fetus probably through a complex genetic predisposition and inter-utero environment interaction, which further “programs” the growth and development pathways that continuously impact the children growth in later life [15]. Excessive maternal weight has also been found to be negatively associated with breastfeeding practices, including initiation, intensity, and weaning before six months [16–18]. Therefore, to understand the influence of the feeding pattern on the growth of infants, maternal weight status should be taken into account. However, to our knowledge, very few studies have been conducted to investigate the interactive impacts of the two on infancy growth indicators. One study from the United States, using overweight and obesity as the outcome variable, concluded that the combination of maternal pre-pregnancy obesity and the lack of breastfeeding might be associated with a greater risk of childhood overweight [19].

The prevalence of overweight and obesity among Chinese women has been rapidly increasing in recent years (from 12% in 1989 to 30% in 2006) [20–22]. Meanwhile, data from the National Program of Action for Child Develop in China 2011–2020 showed a very low EBF rate of 28% in Chinese children during the first six months [23]. A systematic investigation of the joint impact of MPWS and feeding practice on the growth of infants, if any, is needed. To understand the interactive effects of the two on children’s growth is of importance to the health of children, as well as to the early prevention of chronic degenerative diseases in later life.

In this longitudinal study, we aimed to investigate (1) the independent associations of MPWS and early feeding patterns at the first and third months with the offspring’s BMI-Z at ages one and two, and (2) whether the effect of early feeding patterns was altered by MPWS.

## Methods

### Participants

From April 2009 to March 2010, we enrolled 2,220 newborns (1,155 boys and 1,065 girls) in a prospective cohort study from three urban areas in China. Shenyang, Wuhan, and Guangzhou, the capital cities of Liaoning (Northern China), Hubei (Central China), and Guangdong (Southern China) provinces respectively, were selected to represent the three geographic regions of Mainland China. The sample size of 976 was estimated using the simple random sampling method ($n\;=\;{\left(Z_{\alpha /2}\right)}^{2}\times \frac{1}{\varepsilon^{2}}\times \frac{1-P}{P}\;\alpha \;=\;0.05,\varepsilon \;=\;0.15)$, and we used 15% as the estimated rate of overweight and obesity at age two (*P* = 15%). Since it was a cohort study, we used 40% as the estimated rate of lost-to-follow up, the sample size at recruitment was 976/(1–0.4) = 1627. Considering the number of Community Health Service Center (CHSC) and the convenience of quality control, we decided to recruit more children in Wuhan city since the research personnel was based in Wuhan. Five out of 54, ten out of 90 and eight out of 85 CHSCs were selected from these three cities respectively, using a random number table. The information of the eligible infants were obtained from the municipal Maternal and Child Health Information System and the newborns were also randomly selected and thereafter recruited upon the parental informed consents. Given the workload of the following up, each CHSC was required to recruit only 10–15 newborns on a monthly basis. All procedures were standardized across the three field sites and data collectors were centrally trained and monitored.

**Fig 1:**
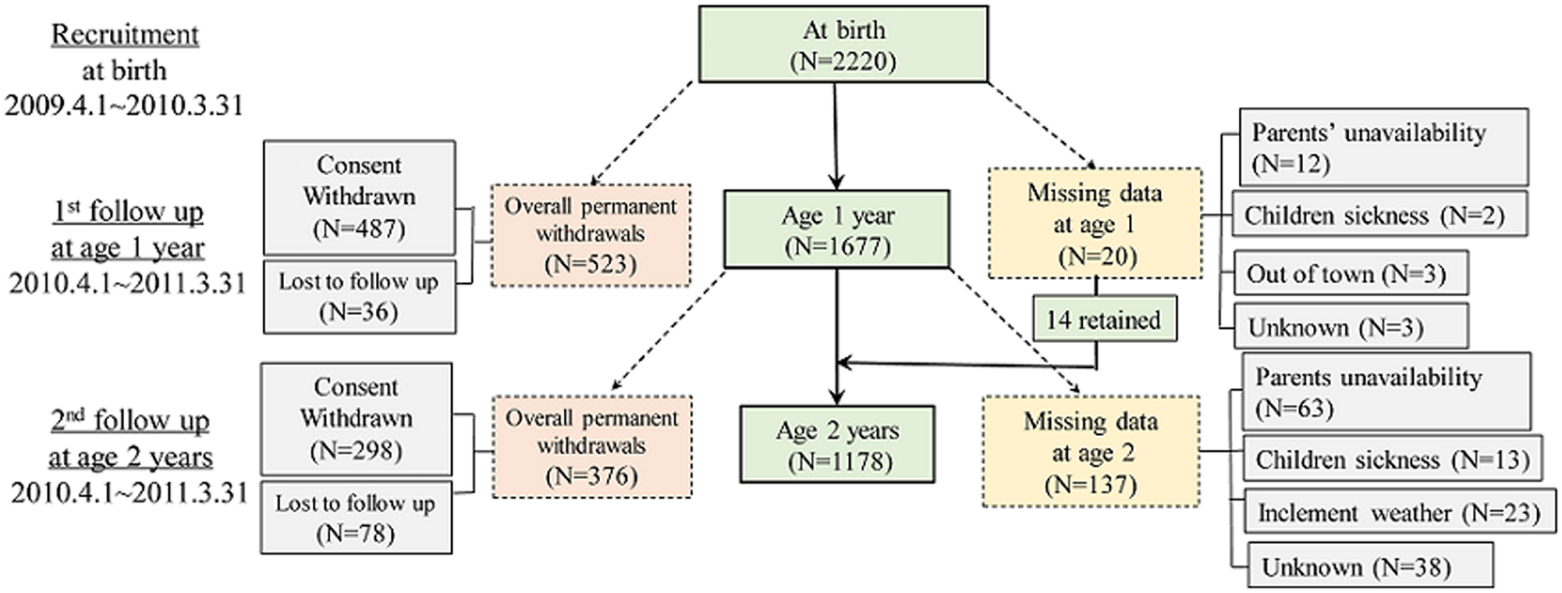
Flow chart of recruitment and loss to following up.

With 2220 participants (565, 1017 and 638 in the three cities) initially recruited at birth, the cohort became 1178 (337, 534 and 307 for the three cities) at the two-year follow up. Fig 1 presents the follow up situation from birth to age two. The overall permanent withdrawals included consent withdrawn and lost to follow up. The missing data resulted from those who could not attend the one year old follow up due to the parents’ unavailability for the clinic visit, children’s sickness, inclement weather, travelling and other reasons. Fourteen out of the 20 families retained at the age-two follow up, missed the one year follow up visit. There was no significant difference detected among the initially recruited and the remaining subjects at one and two-year follow-ups, in terms of their social-demographic variables, children’s birth variables, and parental body mass index (BMI), except for the paternal age. For instance, the parental average age was 31.1 years for all originally recruited participants, compared to 31.3 years for those who were successfully followed up for two years. There was also no difference observed in dropout pattern between the two maternal BMI groups.

### Procedures

Socioeconomic and health-related variables were extracted from the Perinatal Health Booklet (PHB) and from the standardized interviews with parents. Health information, including maternal gestational health status, gestational age, maternal weight gain and mode of delivery, was collected from the PHB, which was self-retained by the parents from the first prenatal checkup until 42 days following delivery. Data on child gender, date of birth, parental age and education level at children’s birth, monthly household income, family structure, and the number of children in the household, smoking and secondhand smoking status during pregnancy were collected at the one-month-old follow-up interview.

Infant birth weight and crown-heel length at birth were measured and recorded on the PHB by the nurses in the delivery room. Weight and length/height were measured within 10 days of the exact month anniversary during the first year, and within 15 days during the second year by the centrally trained child health care staff from CHSC/CHSS. The staff contacted parents by phone two weeks prior to the due day for the measurement as a reminder for the scheduled visit. At one year of age, child body weight and recumbent length were measured with an electronic scale (WHS-I, Wuhan Computer Software Development Co.), and child body weight and standing height was measured with a wall-mounted measuring tape (S-RT-Y1 Healthy kid Intelligent Health Check Wuhan Computer Software Development Co) at age two. Child body weight was measured to the nearest 50g, and both length and height to the nearest 0.1 cm. The measurement was conducted by the centrally trained staff, and the inner-personal variation was tested during training to be under 5%. To further limit the variation of measurement, measurements were taken in duplicate and means of the replicates were used in the analyses. Maternal pre-pregnancy weight and height and those for fathers were self-reported and collected at the one-month follow-up.

Feeding pattern information for the first and third months were collected through face-to-face interviews by the same trained staff at one-month and third-month following-ups. It was originally recorded at each clinic visit by type at each feeding occasion and was categorized into EBF, mixed feeding (MF), and (formula feeding) FF. EBF was defined according to the WHO definition [24]. MF infants were fed with, but not limited to, breast milk and formula milk, while FF infants referred to those fed with formula milk and other food but no breast milk. Actually, only five of FF infants (1.7%) were given fruit juice and/or congee in addition to formula milk at the first month and 32 (7.2%) ate these foods at the third month, in addition to formula milk. Given the limited number of the cases and the minimal amount of other food intake, we did not present exclusively FF data and the FF plus other food data separately.

### Variables

BMI was calculated and used as an indicator of adiposity. Parental weight status was firstly categorized into underweight, normal weight, overweight and obesity, using three BMI cutoffs for Chinese adults, i.e., 18.5 kg/m^2^, 24 kg/m^2^, and 28 kg/m^2^ [25]. But no significant difference effect was found between the maternal underweight and the maternal normal weight on children’s BMI-Z, and as one of our purposes was to assess the high BMI status’s impact on children’s BMI, the underweight and normal weight were combined as UW/NW. Given the small number of obese mothers (1.4%), overweight and obese mothers were combined as OW/OB. Children’ gender and age specific z-scores of BMI was calculated using the WHO Child Growth Standards 2006 [26]. BMI-Z was recalculated through the execution of a SAS macro based on the 2006 WHO growth standards [27].

Preterm birth was defined as birth prior to 37 gestational weeks. The monthly household income (RMB) was recorded into ordinal categories from 1 to 5 (1 = <2,000, 2 = 2,000-<3,000, 3 = 3,000-<5,000, 4 = 5,000-<8,000, and 5 = ≥8,000). Family structure was recorded as four nominal categories, i.e., 1 = nuclear family, 2 = extended family, 3 = joint family (the family consists of 2 or more nuclear families with kinship), and 4 = single-parent family. The feeding pattern was recorded as a three categorical variable, i.e., EBF, MF, and FF. The birth weight was used as a continuous variable.

Due to a small amount of missing data for some variables, the actual number of cases may vary slightly in some descriptive summaries. Variables of children’s birth length, parental age and BMI, and gestational age had less than 15 missing data both at age one (0.89%) and two (1.27%). There were 272 (16.22%) and 273 (16.28%) missing data at age one follow-up for gestational hypertension and diabetes, and the number for the age-two follow-up became 201 (17.06%) and 215 (18.25%) respectively. A total of 121 (7.22%) and 246 (14.67%) records have the missing data in the first and third months’ feeding pattern at age one, and the number of missing data were 75 (6.37%) and 193 (16.38%) at age two.

### Confounders

The confounders were defined based on the literature reports and the results from descriptive analysis of the data. As children’s birth weight and length, and paternal BMI were previously announced to be significantly associated with children BMI-Z, these variables were controlled in the analysis. The social-economic and demographical variables, such as monthly household income and parental age were also controlled for due to the potential impact on parental weight status and children’s feeding practice. The high proportion of extended families in China posts extra risk of being overfed among Chinese children as they were surrounded by more adult in the household. Therefore, family structure was also considered as a confounding variable. Children’s gender was included as the descriptive analysis indicated gender variation—boys appeared to be more EBF than their girl counterparts in the first month (p<0.05).

Variables such as smoking (1.1%), second hand smoking (18.7%), gestational diabetes (1.85%) and hypertension (2.85%), and maternal pregnancy weight gain, delivery mode, and number of children in the household were also reported to have potential impact on the children’s growth, however, were not included in the final models due to the low proportion or insignificant impact on the results.

### Statistical analysis

The database was established on Epidata 3.1 and the descriptive analysis was performed using SAS 9.3. Descriptive characteristics were presented as means and Standard deviation (SD) for continuous variables or as percentages for categorical variables and for maternal UW/NW and OW/OB groups; *t*-test and *chi*-square test were employed to compare the differences respectively. The differences of means on children’s growth measures at age one and two were compared with equal variance *t*-test between maternal weight groups, and with *ANOVA (analysis of variance)* least square deviation test of three feeding groups.

The MPWS and the feeding patterns at the first and third months were the primary exposure. BMI-Z of infants was considered the as outcome variable. General Linear Models (GLM) (cross sectional models) were used to assess the main effects of MPWS and the first and third months’ feeding pattern on BMI-Z at one and two years of age, with the confounders adjusted for. Thereafter, the interactions between MPWS and feeding patterns were assessed with GLM. To assist the interpretation of results, we calculated and graphed the adjusted-means of infants’ BMI-Z at age one and two for the different combination of MPWS and the first and third months’ feeding patterns.

Statistical significance level was set at 0.05.

### Ethics Statement

This study, including the informed consent form, was approved by the Ethics Committee (EC) of Tongji Medical College, Huazhong University of Science and Technology (IORG0003571).

Written informed consent was obtained from the parents of the children involved in this study.

## Results

Table 1 summarizes the characteristics of the subjects based on the two maternal BMI groups and as a total. The OW/OB mothers (8.7%) had on average 6.2 kg/m^2^ of BMI higher than UW/NW mothers. Their offspring were also heavier at birth compared with those with UW/NW mothers (3,460±434g vs. 3,322±416g), with a higher proportion of macrosomia as well (12.9% vs. 6.6%). The difference between OW/OB and UW/NW mothers were also observed in the prevalence of gestational hypertension (10.8% vs. 2.1%), C-section rate (77.8% vs. 67.6%) and the monthly household income (percentage of monthly household income less than 2,000 RMB being 23.2% vs. 14.3%). Meanwhile, OW/OB mothers were less likely to exclusively breastfeed, i.e., only 28.7% of them continued EBF during the first month compared with 37.5% for UW/NW mothers, although the EBF rates were stable in both UW/NW and OW/OB groups from the first month to the third month. The smoking and second-hand smoking rates between the two maternal BMI groups were found to be equal, while the overall second-hand smoking rate was significantly higher than the smoking rate (19.6% vs. 1.7%). No difference was observed in children’s birth length, parental BMI and education level, and the proportions of single child family, family structure and maternal gestational diabetes between the two maternal BMI groups.

**Table 1:** The characteristics of children and family in relation to maternal pre-pregnancy weight status.

Variable	OW/OB mothers (n = 194)	UW/NW mothers (n = 2026)	Total (n = 2220)
Boys [N (%)]*	121 (62.4)	1034 (51.0)	1155 (52.0)
Single child family [N (%)]	171 (88.1)	1766 (87.2)	1937 (87.3)
Birth weight [*mean (SD)*, *g*] *	3460 (434)	3322 (416)	3338 (421)
Birth length [*mean (SD)*, *cm*]	50.5 (1.6)	50.3 (1.7)	50.3 (1.7)
Gestational age [N (%)]			
Preterm	7 (3.7)	73 (3.6)	80 (3.6)
Full term	177 (93.7)	1915 (94.9)	2092 (94.8)
Post term	5 (2.7)	29 (1.4)	34 (1.5)
Delivery mode [N (%)]*			
Vaginal	43 (22.2)	656 (32.4)	699 (31.5)
C-section	151 (77.8)	1370 (67.6)	1521 (68.5)
Maternal age [*mean (SD)*, *year*]*	29.7 (4.3)	28.3 (3.7)	28.5 (3.8)
Paternal age [*mean (SD)*, *year*] *	31.7 (4.7)	31.0 (4.7)	31.1 (4.7)
Maternal BMI [*mean (SD)*, *kg/m* ^*2*^]	26.0 (2.0)	19.8 (1.9)	20.5 (2.6)
Paternal BMI [*mean (SD)*, *kg/m* ^*2*^]	24.3 (3.3)	23.5 (3.3)	23.5 (3.3)
Maternal educational level [N (%)]			
Middle school or less	38 (19.6)	394 (19.5)	432 (19.5)
High school/technical	53 (27.3)	531 (26.2)	584 (26.3)
University/college	101 (52.1)	1016 (50.1)	1117 (50.3)
Master/advanced	2 (1.0)	85 (4.2)	87 (3.9)
Paternal educational level [N (%)]			
Middle school or less	36 (18.6)	307 (15.2)	343 (15.5)
High school/technical	52 (26.8)	592 (29.2)	644 (29.0)
University/college	100 (51.6)	1005 (49.6)	1105 (49.8)
Master/advanced	6 (3.1)	122 (6.0)	128 (5.8)
Monthly income [N (%), *RMB*]*			
<2000	45 (23.2)	289 (14.3)	334 (15.1)
2000-<3000	50 (25.8)	516 (25.5)	566 (25.5)
3000-<5000	64 (33.0)	689 (34.0)	753 (33.9)
5000-<8000	20 (10.3)	361 (17.8)	381 (17.2)
≥8000	15 (7.7)	171 (8.4)	186 (8.4)
Family structure [N (%)]			
Nuclear family	114 (58.8)	1199 (59.2)	1313 (59.2)
Extended family	75 (38.7)	762 (37.6)	837 (37.7)
Joint family	3 (1.6)	57 (2.8)	60 (2.7)
Single-parent family	2 (1.0)	7 (0.4)	9 (0.4)
Maternal smoking [N (%)]	21 (1.4)	7 (4.6)	28 (1.7)
Maternal secondhand smoking [N (%)]	33 (17.0)	402 (19.8)	435 (19.6)
Gestational hypertension [N (%)]*	13 (10.8)	27 (2.1)	40 (2.9)
Gestational diabetes [N (%)]	5 (4.1)	21 (1.6)	26 (1.9)
First month feeding pattern [N (%)]*			
EBF	51 (28.7)	708 (37.5)	759 (36.7)
MF	95 (53.4)	920 (48.7)	1015 (49.1)
FF	32 (18.0)	263 (13.9)	295 (14.3)
Third month feeding pattern [N (%)]			
EBF	44 (27.3)	609 (37.2)	653 (36.3)
MF	69 (42.9)	636 (38.8)	705 (39.2)
FF	48 (29.8)	394 (24.0)	442 (24.6)

* *P*<0.05, significant difference of variables between normal weight and overweight/obese mothers;

Equal variance *t* test was conducted for the corresponding comparison in numerical variables between the two maternal BMI groups; while *Chi-square* test was performed for categorical variables comparison.

The results from the unadjusted comparison, showed in Table 2, indicated that the infants born to OW/OB mothers were significantly greater in BMI-Z at age one and two. However, there was little difference observed in BMI-Z amongst the three feeding groups in first and third months.

**Table 2:** BMI-Z of children at ages one and two according to maternal pre-pregnancy weight status and feeding patterns [*mean (SD)*].

	The first year	The second year
Maternal pre-pregnancy BMI category		
UW/NW	0.60 (0.96)	0.48 (0.95)
OW/OB	0.83 (1.07)*	0.70 (0.92)*
The first month feeding pattern		
EBF	0.58 (0.95)	0.44 (0.91)
MF	0.68 (0.99)	0.51 (0.95)
FF	0.57 (1.01)	0.53 (1.01)
The third month feeding pattern		
EBF	0.61 (0.96)	0.40 (0.95)
MF	0.62 (0.97)	0.51 (0.98)
FF	0.58 (0.94)	0.39 (0.92)

* *P*<0.05, significant difference within age-specified maternal weight status or feeding pattern groups.

No significant main effects of MPWS on children’s BMI-Z at one and two years of age were detected using GLMs, with the confounders adjusted for (age one: *p* = 0.07; age two: *p* = 0.27). Nor was the main effect for feeding pattern at first and third months observed (Table 3).

**Table 3:** The independent effect of maternal weight status and first and third months’ feeding pattern on children’s BMI-Z at age one and age two.

	The first year		The second year	
	Coefficient	*p*	Coefficient	*p*
Maternal pre-pregnancy BMI category		0.07		0.27
UW/NW	*Ref*		*Ref*	
OW/OB	0.17	0.07	0.12	0.27
The first month feeding pattern		0.12		0.19
EBF	*Ref*		*Ref*	
MF	0.14	0.04	0.09	0.22
FF	0.12	0.30	0.24	0.08
The third month feeding pattern		0.52		0.20
EBF	*Ref*		*Ref*	
MF	-0.03	0.66	0.10	0.20
FF	-0.11	0.26	-0.14	0.21

Children’s gender, birth weight and length, paternal BMI, parental age, monthly household income, family structure and recruitment site were adjusted in the models.

Significant interactions of MPWS and feeding patterns on children’s BMI-Z were found, with the adjustment for the indicated variables (Table 4). The number noted in the table was the coefficients for each combination of MPWS and feeding patterns, using EBF infants whom were born to normal weight mothers as the reference group. Children whose mothers were OW/OB and were formula fed at the first month had a significantly greater BMI-Z at ages one and two years (*p*<0.05).

**Table 4:** The interaction between maternal weight status and first and third months’ feeding pattern on children’s BMI-Z at age one and age two.

Feeding pattern		The first year		The second year	
		OW/OB	UW/NW	OW/OB	UW/NW
The first month	FF	0.71*	-0.06	0.60*	0.03
	MF	0.30*	0.09	0.13	0.11
	EBF	-0.22	Ref	0.04	Ref
The third month	FF	0.46*	-0.08	0.33	-0.03
	MF	0.10	0.03	0.09	0.17
	EBF	-0.08	Ref	0.17	Ref

All values (βcoefficients) were generated by GLMs, adjusting for children gender, birth weight and length, monthly household income, paternal BMI and age, and the recruitment site. For instance, compared to the noted reference group, the FF infants born to OW/OB mothers were on average 0.71 units greater in BMI-Z at one year of age.

* Refers to *p*<0.05.

**Fig 2:**
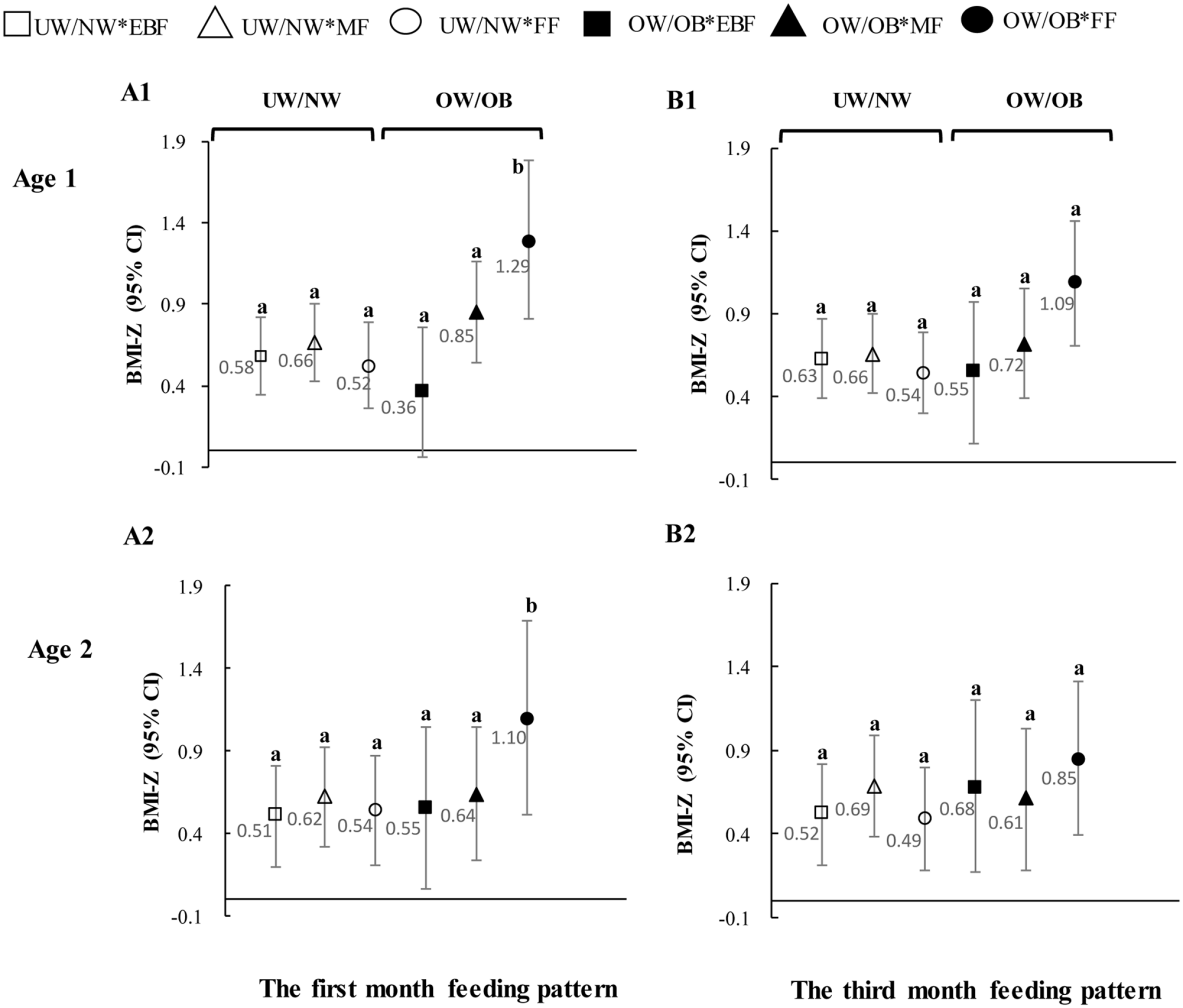
The interactive effects between maternal pre-pregnancy weight status and feeding pattern in the first and third months on children’s BMI-Z at age one and age two.

Fig 2 further shows the adjusted means of BMI-Z and the corresponding confidence intervals (CI) for the six combinations of MPWS and feeding pattern at the first and the third months. The children with OW/OB mothers and were formula fed in the first month had significantly greater BMI-Z than their remaining counterparts at both age one and age two. The corresponding mean BMI-Z was greater than the largest upper limit of the remaining five CIs as shown in Fig 2. The third month FF children with OW/OB mothers also had significant greater BMI-Z than the other counterparts at age one. Graph A1 shows that in the group of OW/OB mothers, the children who were non-EBF (MF and FF) in the first month had significantly greater BMI-Z than their EBF counterparts. The impact of feeding pattern on the children’s BMI-Z was not detected among children whose mothers were UW/NW.

All values were generated by using GLMs, with the adjustment for children gender, birth weight and length, paternal BMI, parental age, monthly household income, family structure and recruitment site. Bars represent 95% CI of corresponding measures.

A1, B1 refer to interaction effects of the first and third months feeding pattern on children’s one-year-old BMI-Z; A2, B2 refer to interaction effects of the first and third months feeding pattern on children’s two-year-old BMI-Z.

## Discussion

In this cohort, 2,220 newborns were initially enrolled in the three cities in China, with 8.7% of the maternal population found to be either overweight or obese, and with an exclusive breastfeeding rate of 36% at both first and third months. We examined the main effects of MPWS and feeding patterns, and their interactive impacts on children’s BMI at one and two years of age. The overall positive association between maternal pre-pregnancy weight status and the offspring BMI-Z observed in previous studies [14], was not confirmed in the current one. Nor was the main effect observed in the feeding pattern. However, the significant interactive effects between the two were clearly revealed from our results. Our study indicated that the complexity of the impact of maternal weight status and early feeding pattern should be acknowledged in order to understand the growth of infants. It further highlighted the importance of retaining a healthy weight prior to pregnancy and promoting exclusive breastfeeding, to reduce excessive growth in BMI of infants. The promotion of EBF is particularly important among those whose mothers were OW/OB before pregnancy [28, 29].

EBF has been reported with the effect of promoting a more desirable growth rate of infants compared with the non-EBF peers [30, 31]. Interestingly, our results demonstrated that those children born to OW/OB mothers and exclusively breastfed in the first months, tended to have a significant lower BMI in contrast with their counterparts who were MF or FF. This association continued up to age two, although the impact on BMI seemed to attenuate at the older age. The infants’ BMI-Z did not alter by feeding patterns if they were born to UW/NW mothers. A similar finding was observed in a study by Li et al, which concluded the additive interaction between maternal pre-pregnancy obesity and the lack of breastfeeding on the risk of overweight among children aged 2–14 [19], although their study used categorical weight status as the outcome variable instead of z-scores as in ours. Some of previous studies, however, reported a contradicting result, which suggested that the offspring of those mothers who are more obese at the time of their pregnancy and thereafter practice breastfeeding, may have been “programmed” to become heavier themselves [32–34]. Nevertheless, early infancy is a critical period for the development of obesity and related conditions [35]. The rapid infant growth from the first week after birth to two years of age is believed to be a predictor of later adiposity [36–39] and a subsequent risk of overweight in the future [40, 41]. Our results appear to support the protective effect of EBF in lowering the potential risk of overweight and obesity in later life, i.e., by reduce the excessive growth in BMI of infants from OW/OB mothers. Differences in serum leptin and ghrelin values in infancy related to feeding pattern [42], the lower energy density in breast milk, and the better self-control of caloric intake in EBF children [43], might explain the variations in the growth indicators between breastfed and formula-fed infants later in life. However, it is not clear yet why this impact only occurs in infants with OW/OB mothers. In our study, we also noticed that maternal weight status showed its impact on their offspring’s growth only when the children were formula fed.

Maternal obesity is becoming a serious public health concern, especially in urban area in China [44]; therefore, it is not difficult to conclude that more and more children will be born to OW/OB mothers. Concurrently, the EBF rate in China is far from ideal. A total of 28% of national EBF rate in the first six months was far lagging behind the target (not less than 50%) set by the National Program of Action for Child Development in China (2011–2020) in 2010 [23, 45]. Figures from the current study have not improved, with an overall EBF rate in the first three months being 36%. This number became 28.7% among OW/OB mothers, which might be associated with late initiation and early termination of breastfeeding [46–48]. Moreover, due to the observed complex interaction between MPWS and feeding pattern on infancy growth in our study, we believe, on the one hand, MPWS should not be omitted in the breastfeeding promotion for its best effectiveness to be accomplished, and its health benefit in infant growth to be maximized. On the other hand, the attention should be attached to the early initiation and maintaining EBF if a longer duration cannot be achieved. This is critical for early childhood obesity prevention, especially among children born to OW/OB mothers, although the BF duration has been the main focus in recent studies [49].

### Limitations

The main limitation of the current study is the relatively short duration of children's follow-up; hence long-term influence of EBF on children growth regarding maternal weight status are yet to be investigated. The definition of mixed feeding might be problematic since the proportion of breast milk verse formula milk had not been taken into consideration. The other potential limitation is the acquisition of children’s birth weight and crown-heel length, and parents’ height and weight via the PHB and self-reported questionnaires. These measures might be relatively imprecise compared to the anthropometric data collected under more carefully controlled research settings. However, some studies have suggested that self-reported weight and height (and the resulting BMI) could be reliable, representative and highly correlated with measured data [50, 51], although the reported data on weight and height may be under estimated [52]. The relatively high level of loss-to-follow-up (25% and 47% for the one and two-year follow up) may also limit generalizability and reduce our power to detect differences between the feeding and MPWS groups. However, we tested the social-economic and demographic variables, birth information, and parental BMI between the initially recruited and remaining participants and no significant differences were detected except for a small difference in the mean age of fathers [53]. No difference in drop out pattern was found between the two maternal BMI groups. The comparative low prevalence (8.7%) of overweight and obesity in our sample is also of concern, compared with the best available figure (17.3%) from women aged 18–39 years from the China Health and Nutrition Survey, in the absence of data from similar population. This variation could result from the inequality in such factors as labor history, sampling site, age, and other factors. Consequently, we believe study attrition should not have appreciable impact on the generalizability of our results.

### Conclusions

The complex interaction between MPWS and infant feeding patterns highlights the importance of controlling body weight prior to pregnancy and well-timed EBF. Consequently, interventions should be focused on the prompt provision of vital information and environmental support to encourage healthy weight status before pregnancy and promote recommended feeding practices afterward [54]. This is to ensure a desirable infant growth rate and to help alleviate the likelihood of obesity in the future, particular for children whose mothers were overweight/ obese before pregnancy.
